# Dr. David S. Smith

**Published:** 1891-06

**Authors:** 


Dr. David S. Smith, of Chicago, died April 29th, 1891, aged seventy-five years. His death occurred on the day following the anniversary of his birth, and was caused by angina pectoris. He was a native of New Jersey, and went to Chicago in 1836, where he has remained ever since. He was reputed to be the pioneer homœopathic physician of the West.

